# News from the Resident Officers' Quarters

**Published:** 1907-04-06

**Authors:** 


### NEWS FROM THE RESIDENT OFFICERS' QUARTERS.

### ST. BARTHOLOMEW'S HOSPITAL.

We learn from the April number of the *St. Bartholomew's Hospital Journal* that the new resident medical officers' quarters, in the first portion of the new out-patient and casualty block of that hospital, are now completed and in occupation. From the detailed account given we gather that these quarters must be quite the best of their kind in London. They appear to fulfil every requirement that could be imagined for the health, comfort, and convenience of the resident medical staff and those who wait upon them. This is exactly as it should be. The governors of this ancient and distinguished foundation have realised that their first duty in reconstructing the hospital is to make proper accommodation for the resident staff, who, up till now, have been very poorly housed. Among the details of these new quarters we note especially the convenient situation, the ample size, and the admirable lighting of the rooms, the modern kitchen equipment, the large number of baths, and the installation of a very complete system of telephones. The furniture of the rooms has been chosen with great care, both with regard to comfort and elegance and to durability. Each officer has a bedroom and a sitting-room, and there is a common room and a handsomely-furnished dining-room. We also note that a squash racquet-court for the medical officers is shortly to be built. The residents at St. Bartholomew's are certainly to be congratulated on the provision that has been made for them in the new buildings, and we commend the action of the governors in this respect. It is peculiarly appropriate that the oldest hospital in London should now have the best and most modern accommodation for its resident medical officers.

